# Stencil Nano Lithography Based on a Nanoscale Polymer Shadow Mask: Towards Organic Nanoelectronics

**DOI:** 10.1038/srep10220

**Published:** 2015-05-11

**Authors:** Hoyeol Yun, Sangwook Kim, Hakseong Kim, Junghyun Lee, Kirstie McAllister, Junhyung Kim, Sengmoon Pyo, Jun Sung Kim, Eleanor E. B. Campbell, Wi Hyoung Lee, Sang Wook Lee

**Affiliations:** 1 Division of Quantum Phases and Devices, School of Physics, Konkuk University, 143-701, Seoul, Korea; 2 Department of Chemistry, Konkuk University, 143-701, Seoul, Korea; 3 Department of Physics, Pohang University of Science and Technology, Pohang, Gyungbuk 790-784, Korea; 4 EaStCHEM, School of Chemistry, University of Edinburgh, David Brewster Road, Edinburgh EH9 3FJ, United Kingdom; 5 Department of Organic and Nano System Engineering, Konkuk University, 143-701, Seoul, Korea

## Abstract

A stencil lithography technique has been developed to fabricate organic-material-based electronic devices with sub-micron resolution. Suspended polymethylmethacrylate (PMMA) membranes were used as shadow masks for defining organic channels and top electrodes. Arrays of pentacene field effect transistors (FETs) with various channel lengths from 50 μm down to 500 nm were successfully produced from the same batch using this technique. Electrical transport measurements showed that the electrical contacts of all devices were stable and the normalized contact resistances were much lower than previously studied organic FETs. Scaling effects, originating from the bulk space charge current, were investigated by analyzing the channel-length-dependent mobility and hysteresis behaviors. This novel lithography method provides a reliable means for studying the fundamental transport properties of organic materials at the nanoscale as well as enabling potential applications requiring the fabrication of integrated organic nanoelectronic devices.

Top contact approaches, which deposit source/drain electrodes on semiconductor channel regions, using complementary metal–oxide–semiconductor (CMOS) technology have been widely applied to fabricate and investigate various nanoscale electronic systems. However, it is regarded as problematic to use this approach to achieve the reliable fabrication of organic-materials-based nanoelectronic devices and their integration because of their intolerance towards organic solvents used in standard fabrication procedures. For this reason, there have been only a few trials of the top contact approach applied to micro-scale organic devices for investigating their electrical properties^1^,^2^,^3^,^4^,^5^,^6^,^7^ even though the top contact approach provides superior electrical performance compared to the alternative, bottom contact approach where the semiconductor channel is deposited on top of the source/drain electrodes^8^,^9^.

Various fabrication techniques for pursuing the down-scaling of organic electronic devices using the top contact approach have been suggested such as polydimethylsiloxane (PDMS) stamping 10,11, the use of a SiN shadow mask prepared by a complex top-down fabrication process^1^,^12^,^13^,^14^ and inkjet printing methods^15^. The transfer technique using a PDMS stamp provides the direct deposition of electrode patterns on organic materials. However, this method has only been adopted for large-scale pre-deposited organic thin films so far but has not been applied to nanoscale patterned organic materials with various sizes and dimensions. An etched SiN shadow mask was suggested for producing sub-micron scale organic transistors and has been used to fabricate small-molecule organic semiconductor devices^16^,^17^,^18^,^19^. Despite the high resolution possible with a SiN mask, it is difficult to align electrodes on top of pre-deposited nanoscale organic material. This, together with the indistinct edge of the electrode caused by the diffusion of metal through the air gap between the mask and target substrate, are fundamental limitations of this technique. A recently developed inkjet printing technique showed the possibility of manufacturing organic light-emitting diodes (OLEDs) on a flexible substrate^20^ and realization of sub-micron channel organic transistors with top contact electrodes^15^. However, the top contact approach using inkjet printing is often limited by the ink solvent which causes damage to the organic active layer^21^. Therefore, even though the previously suggested methods have shown promising potential for the fabrication of organic nanoelectronic devices, each of the processes still has some drawbacks and, consequently, the development of a novel fabrication method that overcomes these drawbacks is desired.

In this work, we demonstrate a simple stencil lithography technique using a polymer shadow mask for fabricating nanoscale organic electronic devices. The active organic channel layer and the top electrodes could be clearly defined using standard electron beam lithography processes and precisely positioned by micro-manipulation of shadow masks. Metal diffusion between the stencil mask and substrate, which can lead to poor definition of deposited structures, was prevented by having a gapless contact between the shadow mask and the target substrate. The quality of the organic layer was protected by using mechanical lift-off without introducing any organic solvents during the device fabrication procedure. An array of nanoscale pentacene-stripe field effect transistors, which have channel lengths varying from 50 μm to 500 nm was produced in one fabrication run. The channel-length-dependence of transistor performance was measured and the scaling effects on mobility and hysteresis were investigated. Our technique allows the reliable fabrication of individual organic nanoelectronic devices and also opens up the possibility to realize integrated organic nanoelectronic systems.

## Experimental

### Nanoscale polymer shadow mask preparation and device fabrication

**Figure 1:**
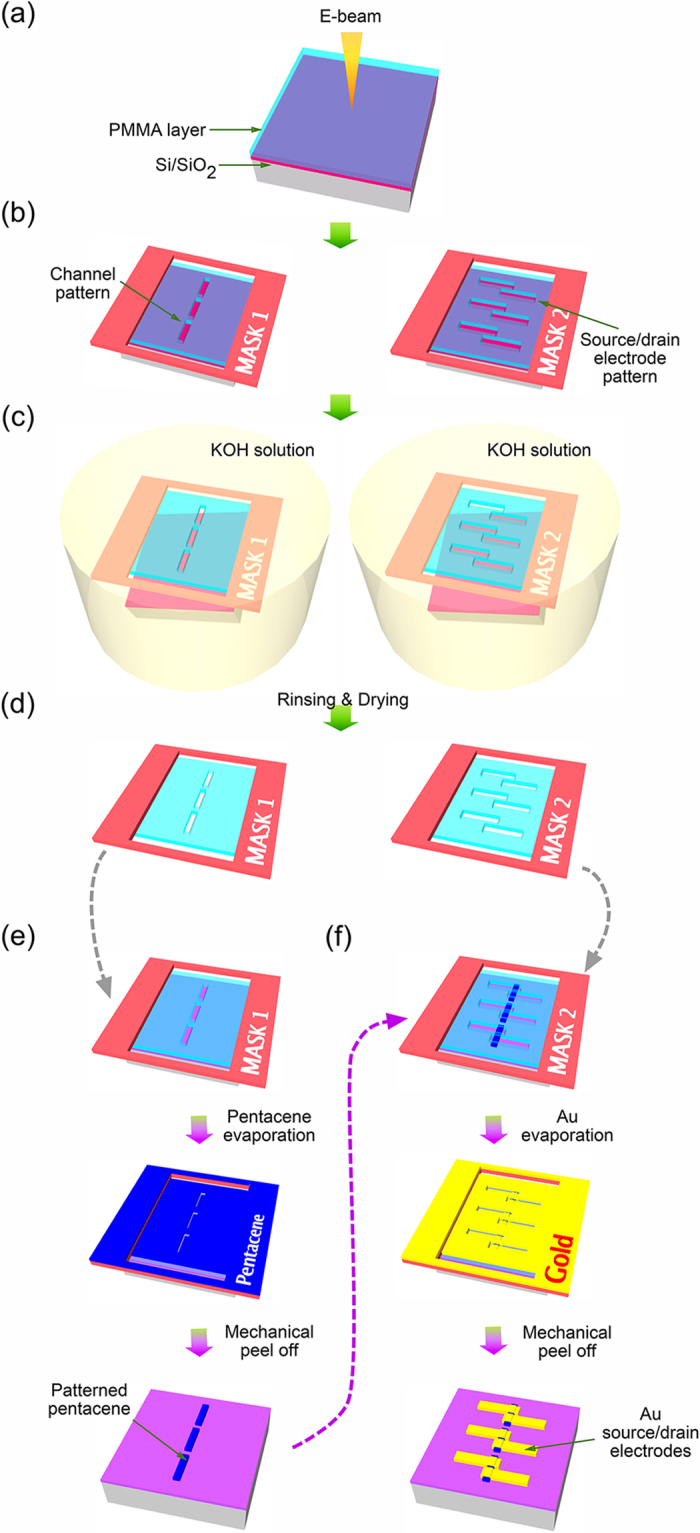
The production of nanoscale polymer shadow mask and fabrication procedure for pentacene based field effect transistor. (**a**) The PMMA layer was spin-coated at 4500 rpm on Si/SiO_2_ substrate and baked during 1 hour on the hot plate at 180 °C (**b**) The patterns for organic channels and source-drain electrodes were developed and the plastic holder was attached on top of the PMMA layer. (**c**) The PMMA layer with holder was separated from the substrate in 4M KOH solution (at 50 °C for 30 min). (**d**) After rinsing with DI-water and drying with N_2_ flow, the shadow masks were ready to be used for defining pentacene channels and subsequent electrode evaporation. (**e**) Mask 1 for pentacene deposition was attached to the Si/SiO_2_(90 nm)/OTS substrate and a 50 nm thick pentacene layer was deposited by thermal evaporation at a deposition rate of 0.1 Å/s. Pentacene micro stripe patterns were defined after mask 1 was mechanically peeled off. (**f**) Mask 2 (for electrode evaporation) was aligned onto the pentacene stripes using a home-made micromanipulator. 70 nm thick Au was deposited using a thermal evaporator under vacuum conditions (~10^−6^ Torr). The fabrication of the organic semiconductor field effect transistors was completed after mask 2 was mechanically detached.

The required pattern for the nanoscale stencil lithography was prepared using electron beam lithography on polymethylmethacrylate (PMMA) spin-coated onto a Si/SiO_2_ substrate, as shown in Fig. 1a. It is necessary to prepare a relatively thick and robust layer of PMMA to prevent the disruption of the mask pattern during the subsequent fabrication steps. For this reason, a 500 nm thick double layer of PMMA was deposited at a spin-coating speed of 4500 rpm. It was also found that an excessive baking time of 1 hour at 180 ^o^C gives higher stability and endurance of the PMMA shadow mask during device fabrication. A piece of thin plastic sheet (Thermal release tape, Nitto Denko, No3198MS), which contains a hole in the center to expose the pattern of the electrodes and pentacene channels, was attached to handle the PMMA membrane in a safe and controllable manner as shown in Fig. 1b. The patterned PMMA layer with the plastic holder was separated from the Si/SiO_2_ substrate in 4 M potassium hydroxide (KOH) solution (Fig. 1c). It is well known that KOH is an effective etchant for Si but is rather inefficient for SiO_2_. The PMMA membrane containing the nanoscale apertures of the electrode patterns remained intact due to the mild and slow etching of the surface of the SiO_2_ layer underneath the PMMA mask by the KOH solution. The PMMA layer with holder was completely released from the Si/SiO_2_ substrate after 30 min in KOH solution at ~50 ^°^C. After rinsing using de-ionized water, the suspended PMMA shadow masks with channels for pentacene deposition (denoted by Mask 1) or electrode deposition (denoted by Mask 2) were completed as shown in Fig. 1d.

The pentacene channels were defined using the stencil lithography method as shown in Fig. 1e. Mask 1, with the dashed line pattern corresponding to the shape of the pentacene channels was attached to a new Si/SiO_2_ substrate. The substrate was pre-treated with octadecyltrichlorosilane (OTS) to planarize the surface of the SiO_2_ layer^22^ before pentacene deposition and to reduce interfacial trap states at the position of the pentacene channels, thus improving device performance. A 50 nm thick pentacene layer was deposited at a deposition rate of 0.1 Å/s on the substrate by thermal evaporation. Mask 1 was then mechanically peeled-off, leaving microstripes of pentacene channels on the substrate. To connect electrodes to the pentacene layer, mask 2 was placed onto the patterned pentacene. The apertures representing the source and drain electrodes were overlapped with the pentacene channels. A gold layer of 70 nm thickness was deposited through the shadow mask using a thermal evaporator so that each channel of the pentacene array was provided with a pair of electrodes, each with a different spacing, thus providing devices with different channel lengths. The fabrication of the pentacene field effect transistors (FETs) was completed after mask 2 was mechanically peeled off. (Fig. 1f).

**Figure 2:**
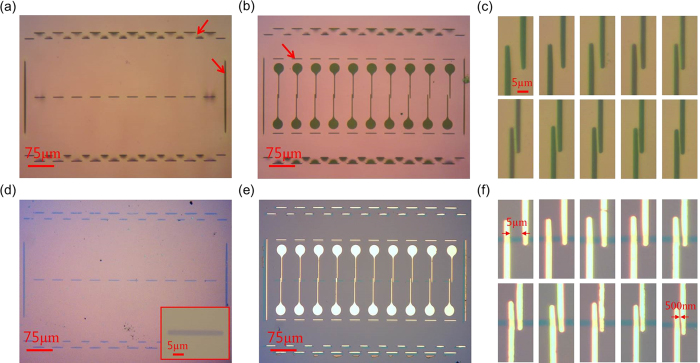
Optical microscope images of fabricated shadow masks and pentacene devices. The shadow masks for (**a**) deposition of pentacene layer (mask 1) and (**b**) electrode deposition on pentacene micro strips (mask 2). (**c**) Enlarged channel parts of the mask of (**b**). The distance between apertures was varied from 5 μm to 500 nm. (**d**) Patterned pentacene layer using mask 1. The inset shows an enlarged image of a pentacene strip. (**e**) Completed pentacene field effect transistors following gold electrode deposition using mask 2 and mechanically peeling off the mask. (**f**) Zoomed-in image of pentacene FETs with different channel lengths.

Figures 2a,b show optical microscope images of mask 1, for deposition of the pentacene layer, and mask 2, for deposition of source-drain electrodes on top of the channels, respectively. These images show that there is no significant deformation in any direction of the released PMMA membrane. An array of apertures was defined at the center of mask 1. Each aperture for the pentacene channels has 30 μm length and 2.5 μm width. The size and aspect ratio of the apertures were precisely reproduced on deposition through the mask. In the case of mask 2, source and drain electrode apertures were interdigitated at the center of the mask and the distances between the two apertures were varied from 5 μm down to 500 nm as shown in Fig. 2c. Circular micro-contact pads were patterned at the end of the source and drain electrodes.

In addition to the channel and electrode patterns in the central area, additional “stitch” apertures were designed at the side of the main pattern on the masks and also close to the contact pad areas as indicated with red arrows in Fig. 2a,b. These stitch apertures relieved the residual stresses of the PMMA, which arose after the PMMA membranes were separated from their initial substrate. It was found that the designed stitches significantly reduced the residual stresses and, consequently, the apertures of the main patterns on the masks could sustain their original configurations without any distortion even when the mask was mechanically peeled off after material deposition. For the same reason, the edges of all the apertures were rounded to avoid any unexpected tearing or cracking at the edge of the pattern area.

Figure 2d is an optical microscope image of the deposited pentacene microchannels. As mentioned above, the dimensions of the evaporated pentacene channels exactly followed the original mask design. No polymer residue was found on the substrate after mask 1 was mechanically peeled off. Fig. 2e shows the completed pentacene FETs after the electrode patterns were deposited by stencil lithography using mask 2. Since the shadow mask is transparent, the pre-defined pentacene channels can be seen through mask 2 so that the position of the electrodes on the pentacene could be controlled accurately. Using a home-made mask alignment system, consisting of a micro-manipulator with an optical microscope, all of the 10 pairs of electrodes were placed on top of each pre-deposited pentacene channel in one lithography step, as shown in Fig. 2f. The channel lengths of the FETs shown in Fig. 2 vary from 5 μm to 1 μm with 1 μm variation for the first 5 devices and vary from 900 nm to 500 nm with 100 nm variation for the remaining ones.

## Results and discussion

**Figure 3:**
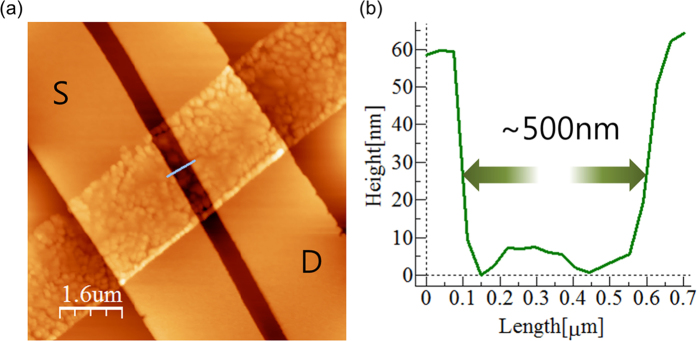
AFM analysis for the resolution of stencil nano lithography. (**a**) Atomic force microscope image for the pentacene FET with 500 nm channel length. (**b**) Cross-section profile of the channel along the grey line (perpendicular to the electrodes) in (**a**).

Figure 3a shows an atomic force microscopy (AFM) topography image of the pentacene FET device with 500 nm channel length. The AFM cross-section profile (Fig. 3b) confirms that the deposited electrode edge is clearly defined and the channel length corresponds well to the distance between the apertures of the source-drain electrode patterns on mask 2. The gapless contact between the masks and the substrate allows an accurate and clearly defined deposition of organic material and metal. It is notable that all of the pentacene channels retained their original shape without being damaged by the mechanical peeling off of the second stencil lithography mask for the top electrode deposition. It seems that the adhesion at the interface between the PMMA layer and the pentacene was not strong enough to impart any physical damage to the deposited organic material, even although the PMMA layer was attached directly on top of the pre-defined channel. Since no chemical solvent was applied during the entire device fabrication process, the method is also chemically non-destructive towards the organic structure. The reusability of the mask was also confirmed by the repeated deposition of pentacene and gold patterns on the same substrate. (See Supplementary Information). Since the PMMA mask becomes opaque after metal evaporation, the stitches and larger apertures, patterned at the outer area of the masks can be used as alignment markers for additional evaporations. In addition, this method is also cost effective and utilizes simpler processes to realize organic nanoelectronic devices compared to previously reported studies^16^,^17^,^18^,^19^.

**Figure 4:**
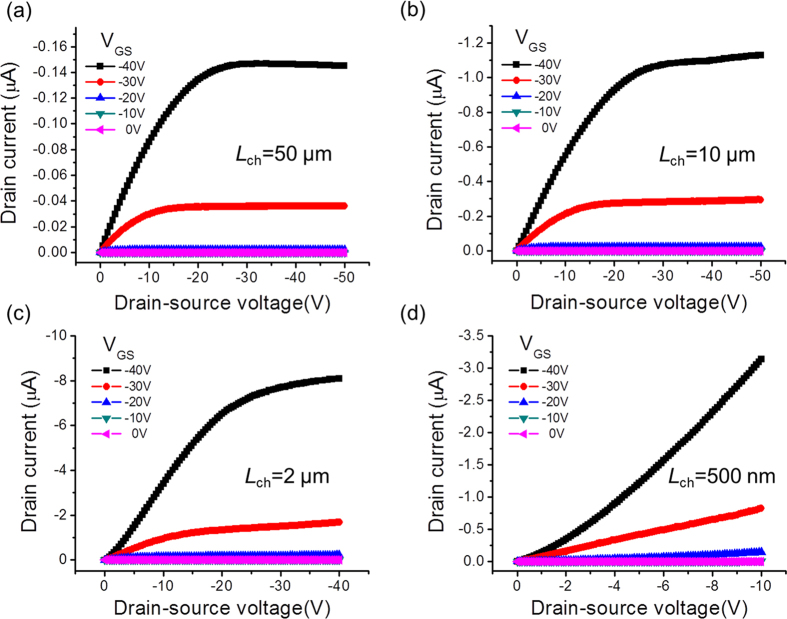
I_D_-V_DS_ characteristics of pentacene FETs with selected channel lengths. The applied back gate voltage (V_GS_) was varied from -40 V to 0 V with 10 V intervals. The channel width of all transistors was 2.5 μm.

Figure 4 shows the drain current-voltage (I_D_-V_DS_) characteristics of the pentacene FETs with various channel lengths (*L*_*ch*_). The measurements were carried out with an HP 4145B parameter analyzer at room temperature and under ambient conditions. Four representative I_D_-V_DS_ curves were chosen to show the channel length dependence of FET performance. It should be noted that all of the pentacene FETs used in these studies were prepared using identical fabrication conditions. The experimental results discussed below were obtained from the same array of pentacene FETs unless otherwise noted. The output characteristics of all other FETs with different channel lengths are presented in the Supplementary Information to illustrate the gradual transition of the electrical performances. For larger channel lengths, longer than 10 μm, the I_D_-V_DS_ curves follow standard FET behavior with a clear saturation region. As the channel length of the FETs is decreased, the linear region of the I_D_-V_DS_ curves is extended and the drain currents are not perfectly saturated. In the case of a 500 nm channel length, the FET device does not show standard FET saturation performance any longer and instead, shows continuous growth of the current value as V_DS_ increases until breakdown occurs. A non-linear I_D_-V_DS_ region appears at low bias voltage when the channel length is decreased to around 2 μm and this non-linearity becomes more prominent as the channel length decreases further. This non-linear behavior, especially for a top contact configuration, in the I-V characteristics is known to originate from charge transfer from the contact area to the channel edge^22^. In the case of FETs with relatively long channel lengths, this effect is screened since the channel resistance is much higher than the contact resistance. It seems that the channel resistances become comparable to the contact resistances for pentacene FETs with channel lengths below 2 μm. Therefore, the influence of the contact resistance becomes increasingly important as the channel length decreases^1^,^23^,^24^,^25^. The detailed analysis of the contact resistance of our devices will be discussed below.

**Figure 5:**
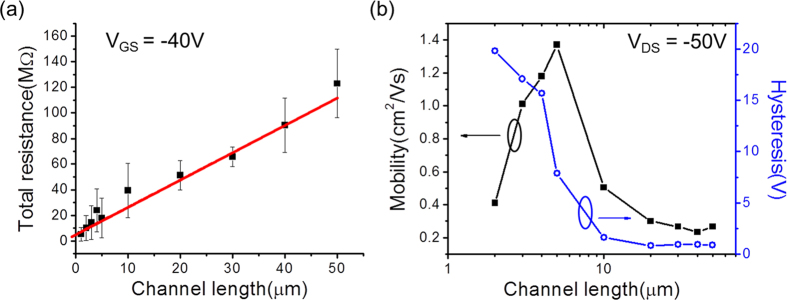
Overview of channel length-related electrical performances. (**a**) Channel length dependence of the total resistance estimated from the output characteristics with –40 V of gate voltage. The red line shows a linear fit. (**b**) The saturation mobility (filled squares) and hysteresis (empty circles) estimated from the transfer curves obtained at –50 V of source-drain voltage with various channel lengths.

The channel-length-dependent total resistances of the pentacene FETs are displayed in Fig. 5a. The resistances were estimated from the I-V curves at a gate voltage (V_GS_) of –40 V for each channel length, from 1 μm to 50 μm. To extract the contact resistance between the pentacene and the electrode, we assumed that the resistivity of the pentacene is uniform along the channel lengths. Also, the contact resistances of the electrode contact areas of all the FETs are assumed to be the same. The measured total resistances indicate the series resistances composed of the two contact resistances and the channel resistance. By using the conventional transfer length method with above conditions, the contact resistance of the pentacene FETs was estimated to be R_C_ = 5.25 ± 1.82 MΩ. The width-normalized contact value after considering the dimension of the contact between the electrode and pentacene is R_C_·W = 1.313 ± 0.455 kΩcm. This value is at least 100 times lower than previously reported values from pentacene devices based on the bottom contact method using pre-deposited bottom electrodes without a self-assembled monolayer^26^,^27^, and is also comparable to or even lower than previous results based on top contact measurements^2^,^10^. The results for devices with sub-micrometer channel lengths could not be used to analyze the contact resistance using the transfer length method because it was difficult to determine the total resistance of these devices due to the strong non-linearity of the I-V characteristics. Moreover, no clear tendency of the current value changes according to the channel length variations was found in this region. This is considered to be related to the grain size of the evaporated pentacene layer. Since the channel length of the FET becomes comparable to the grain size of the pentacene as it is reduced down to the submicron scale, the channel resistance could be sensitively changed according to the morphology of the channel defined by the electrode fabrication.

The mobility and hysteresis of pentacene FETs, estimated by measuring the transfer characteristics with various channel lengths are summarized in Fig. 5b. Representative transfer characteristics of the pentacene FETs are displayed in the Supplementary information. Since the saturation behavior of the output characteristics was suppressed at the submicron scale, meaningful saturation mobility values could only be extracted for devices with channel lengths above 2 μm. The calculated mobility values are around 0.267 ± 0.027 cm^2^/Vs without large variations in the channel length range from 50 μm down to 20 μm. As the channel length is decreased beyond 20 μm , the mobility increases, reaching a maximum of 1.37 cm^2^/Vs at 5 μm before decreasing to 0.41 cm^2^/Vs for a channel length of 2 μm. The origin of this trend for the channel length dependence of the estimated mobility can be rationalized as follows. On the one hand, space charge limited current (SCLC) is expected to increase as the channel length is decreased^28^,^29^,^30^ and, consequently, the measured total current and estimated mobility would be expected to be higher for the shorter channel devices than for the longer ones. On the other hand, the gate electric field will be less effective as the channel length is decreased due to the device geometry. When the gate oxide thickness is comparable to the channel length, the gate field can be masked by the source-drain bias so that the device becomes insensitive to gate voltage changes^30^,^31^,^32^. It can be explained that, in the case of our device structure, the maximum estimated mobility value at a channel length of 5 μm is the result of the competition between these two effects. A similar trend of the scaling effects of the channel length on the mobility could be found in the linear regime, even although the absolute value of the linear mobility is lower than the saturation mobility. All results on the saturation mobility values for microscale channels and the linear mobility values of all devices, including the sub-micron scale channels, are summarized in the Supplementary Information.

The hysteresis of the transfer characteristics also varies as a function of the channel length. The hysteresis values were found to be around 1 V within the range of 20 – 50 μm channel lengths and then increased for the shorter channel lengths from 10 μm downwards. Interestingly, the increase of the hysteresis and the increase of the mobility occurred for the same channel lengths, at 10 μm. While the mobility decreased again for channel lengths shorter than 5 μm, the hysteresis continued to increase as the channel length was reduced further. It is known that clockwise hysteresis in the transfer characteristics, as shown for the present measurements, mainly originates from charge traps at the semiconductor-insulator interface or in the bulk region of the semiconductor^33^,^34^,^35^. In the case of the devices studied here, the interface-induced charge traps are not expected to significantly influence the channel length dependence of the hysteresis since the absolute trap density at the interface should be uniform along the channel. Moreover, charge traps at the interface could be effectively removed by using OTS treatment during device fabrication^36^,^37^. However, bulk trap sites due to crystalline defects or grain boundaries in the pentacene can still exist. As the channel length decreases, the contribution of SCLC conduction can dominate the channel currents so that these bulk traps can dominate the hysteresis behavior. Therefore, the shorter the channel lengths are, the larger is the hysteresis. This explanation provides a qualitative understanding of the increase in both mobility and hysteresis for the same channel length, at around 10 μm.

## Conclusion

In this study, nanoscale stencil lithography based on the use of a PMMA shadow mask was developed. The suitability of the technique for fabricating nanoscale organic electronic devices with top contacts is demonstrated by the presentation of results on the electrical properties of pentacene devices with a range of accurately defined channel lengths. The solvent-free fabrication process avoided any chemical damage to the pentacene and there was no evidence of mechanical damage caused by mechanical peeling of the shadow mask. The electrical measurements on the pentacene FETs showed reliable contact properties with relatively low normalized contact resistances. The channel length dependent mobility of the FETs and the hysteresis in their transfer characteristics was studied. It was found that SCLC dominates for channel lengths below ca. 10 μm.

We have presented a novel fabrication method that can accurately and reliably produce micro- and nano-scale electronic devices from sensitive organic materials. The technique can be easily adapted to realize practical organic-material-based integrated nanoelectronic systems.

## Author Contributions

H.Y., S.W.L., and J.S.K. designed and interpreted the experiments. H.Y., S.K., H.K., J.L. and J.K. performed the experiments and analyzed the data. S.W.L., E.E.B.C., S.P. and W.H.L. provided advices on experimental design and data evaluation. H.Y.,K.M., E.E.B.C. and S.W.L. wrote the manuscript. All authors discussed the results.

## Additional Information

**How to cite this article**: Yun, H. *et al*. Stencil Nano Lithography Based on a Nanoscale Polymer Shadow Mask: Towards Organic Nanoelectronics. *Sci. Rep.* **5**, 10220; doi: 10.1038/srep10220 (2015).

## Supplementary Material

Supplementary Information
