# Supplementary material for: Stencil Nano Lithography Based on a Nanoscale Polymer Shadow Mask: Towards Organic Nanoelectronics
Source: Sci Rep. 2015 May 11;5:10220. doi: 10.1038/srep10220 (PMC4426698; doi:10.1038/srep10220)
Supplement: Supplementary Information [file srep10220-s1.doc]

Supplementary Information

Stencil Nano Lithography Based on a Nanoscale Polymer Shadow Mask: Towards Organic Nanoelectronics

Hoyeol Yun, Sangwook Kim, Hakseong Kim, Junghyun Lee, Kirstie McAllister, Junhyung Kim, Sengmoon Pyo, Jun Sung Kim, Eleanor E. B. Campbell, Wi Hyoung Lee and Sang Wook Lee*


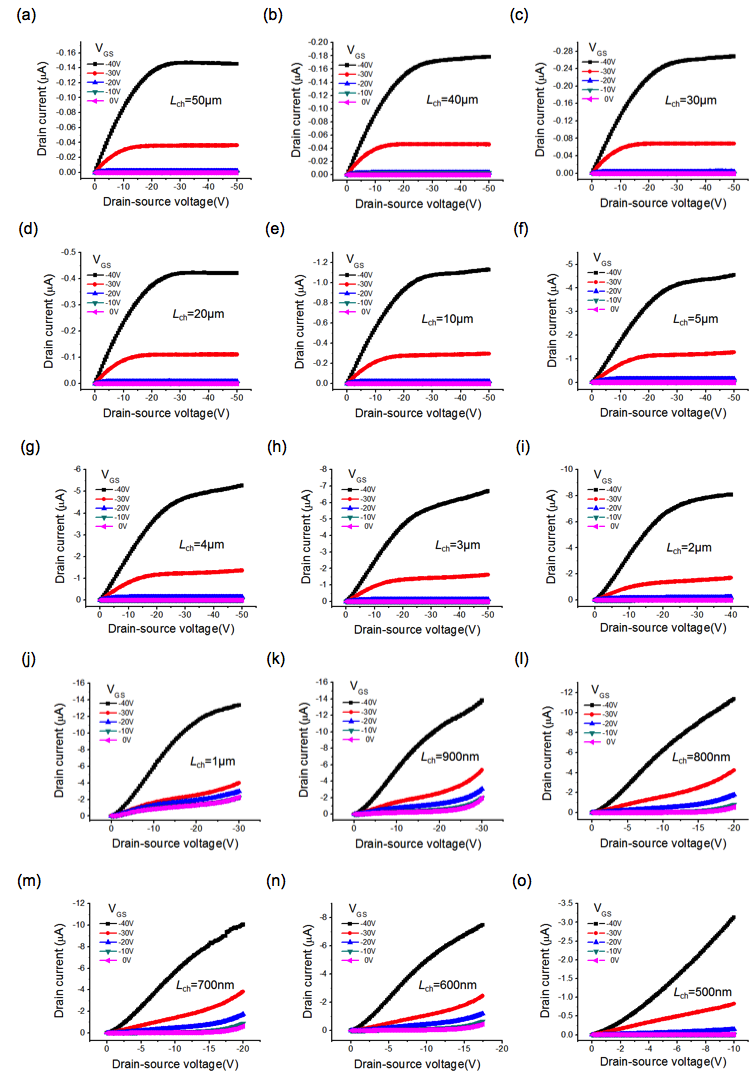


**Supplementary Figure 1**. **The output characteristics of pentacene FETs for all prepared channel lengths.** The channel length was varied with 10 µm spacing in the range of 50 µm (a) to 10 µm (e) and the interval of channel length was decreased to 1 µm from 5 µm (f) to 1 µm (j). For the sub-micron region, from (k) to (o), the channel lengths were decreased down to 500 nm by 100 nm steps.


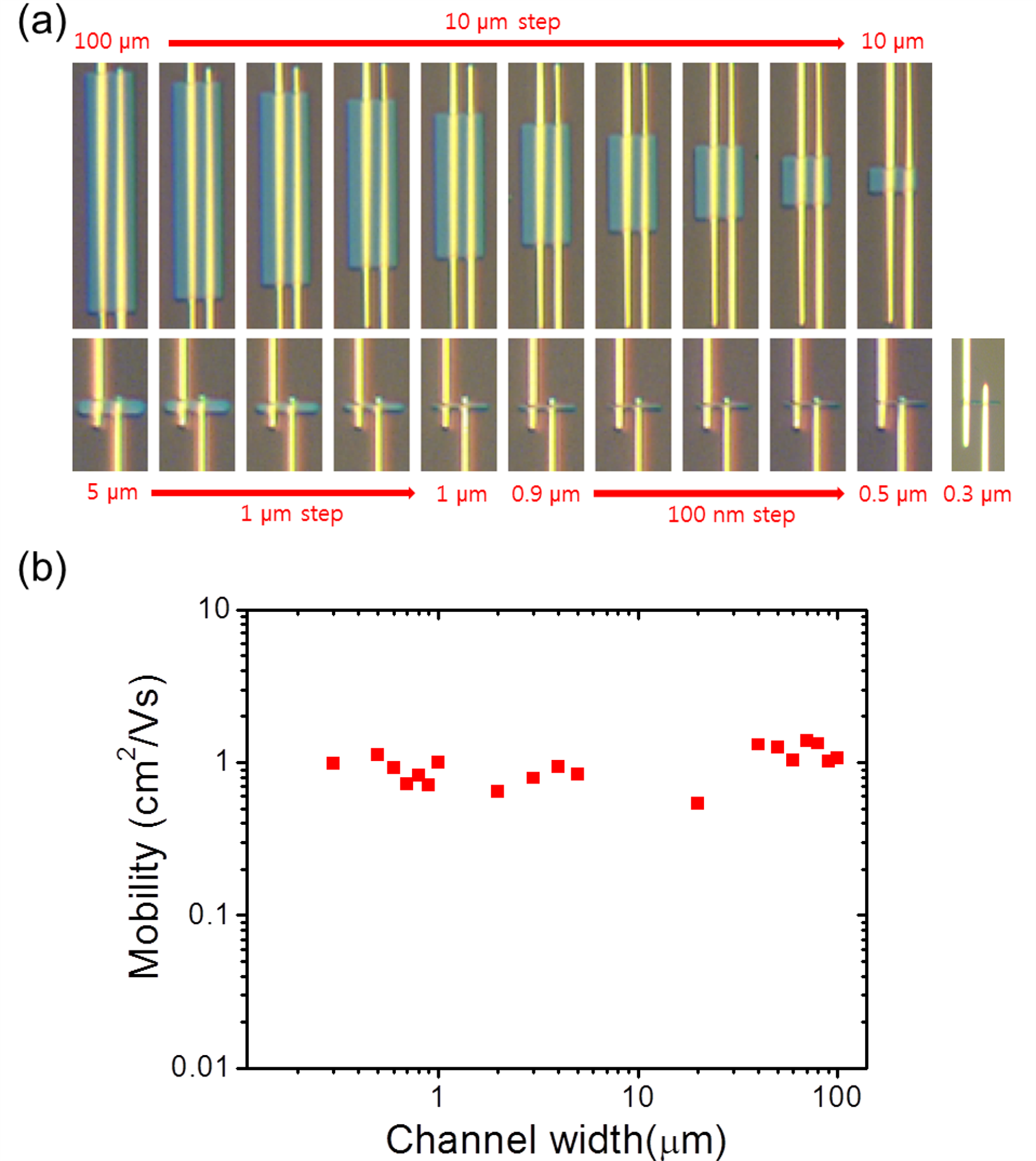


**Supplementary Figure 2**. **Channel width varied pentacene FETs and their electrical performance.** (a) The optical images for pentacene FETs with controlled channel widths. The widths were varied from 100 µm down to 300 nm while the channel length was fixed to 5 µm. (b) Channel width dependence of the field effect mobility. The mobility values are distributed around 1 cm^2^/Vs with no significant dependence on channel width.


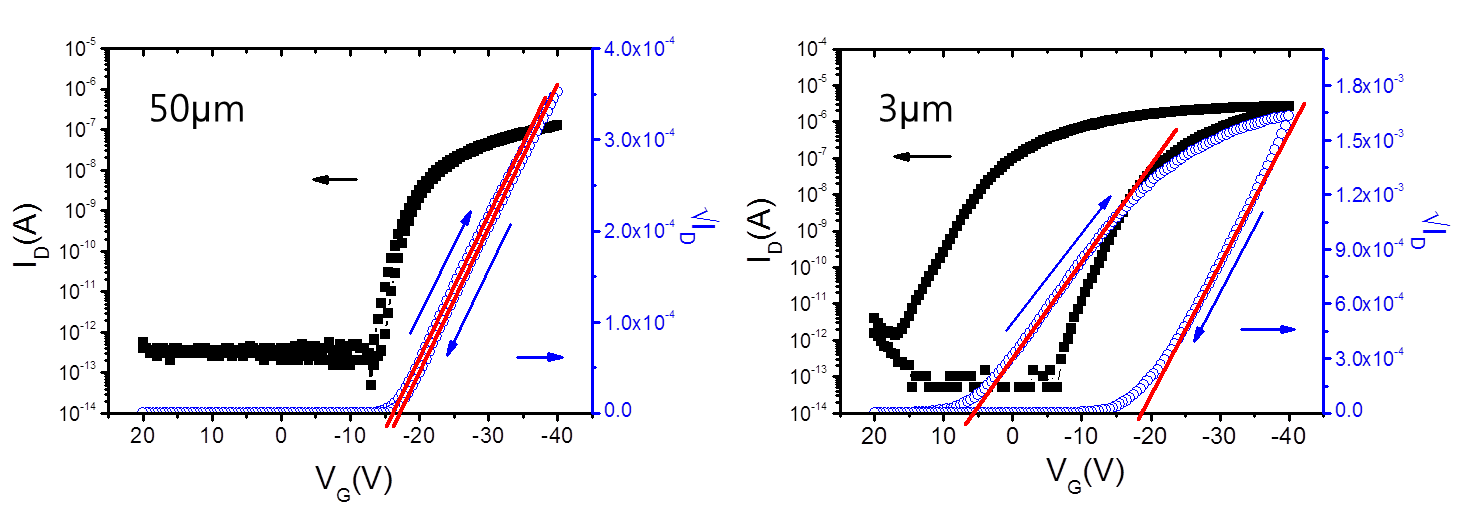


**Supplementary Figure 3**. **The transfer characteristics of a pentacene FET with 50 µm and 3 µm channel lengths.**

**
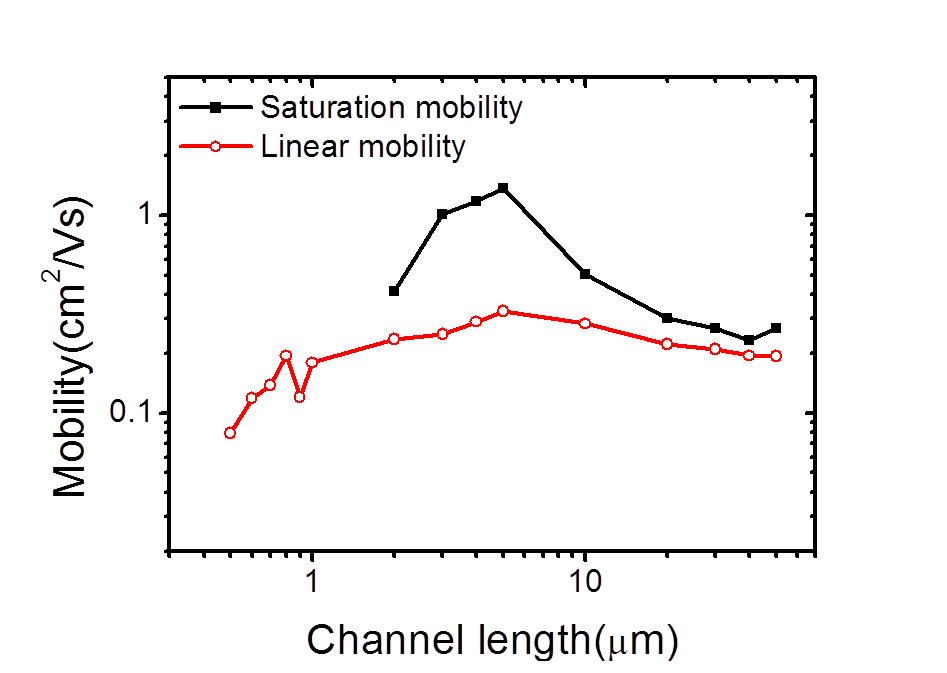
**

**Supplementary Figure 4**. **The field effect mobility of pentacene FETs in saturation and linear regime**

**Supplementary Table 1**. **The mobility values for saturation and linear regime**

| Channel lengths | µ_sat_ (cm^2^/Vs) | µ_lin_ (cm^2^/Vs) | on/off |
| --- | --- | --- | --- |
| 50 µm | 0.267 | 0.194 | 3.4×10^5^ |
| 40 µm | 0.234 | 0.195 | 1.64×10^6^ |
| 30 µm | 0.267 | 0.21 | 1.6×10^6^ |
| 20 µm | 0.3 | 0.222 | 2.26×10^6^ |
| 10 µm | 0.504 | 0.283 | 2,98×10^6^ |
| 5 µm | 1.37 | 0.327 | 2.66×10^8^ |
| 4 µm | 1.18 | 0.289 | 1.58×10^8^ |
| 3 µm | 1.01 | 0.249 | 9.7×10^7^ |
| 2 µm | 0.41 | 0.235 | 1.71×10^8^ |
| 1 µm | - | 0.18 | 5.3×10^7^ |
| 900 nm | - | 0.12 | 5.6×10^7^ |
| 800 nm | - | 0.195 | 3.26×10^7^ |
| 700 nm | - | 0.138 | 6.9×10^7^ |
| 600 nm | - | 0.119 | 2.54×10^5^ |
| 500 nm | - | 0.079 | 7.92×10^5^ |


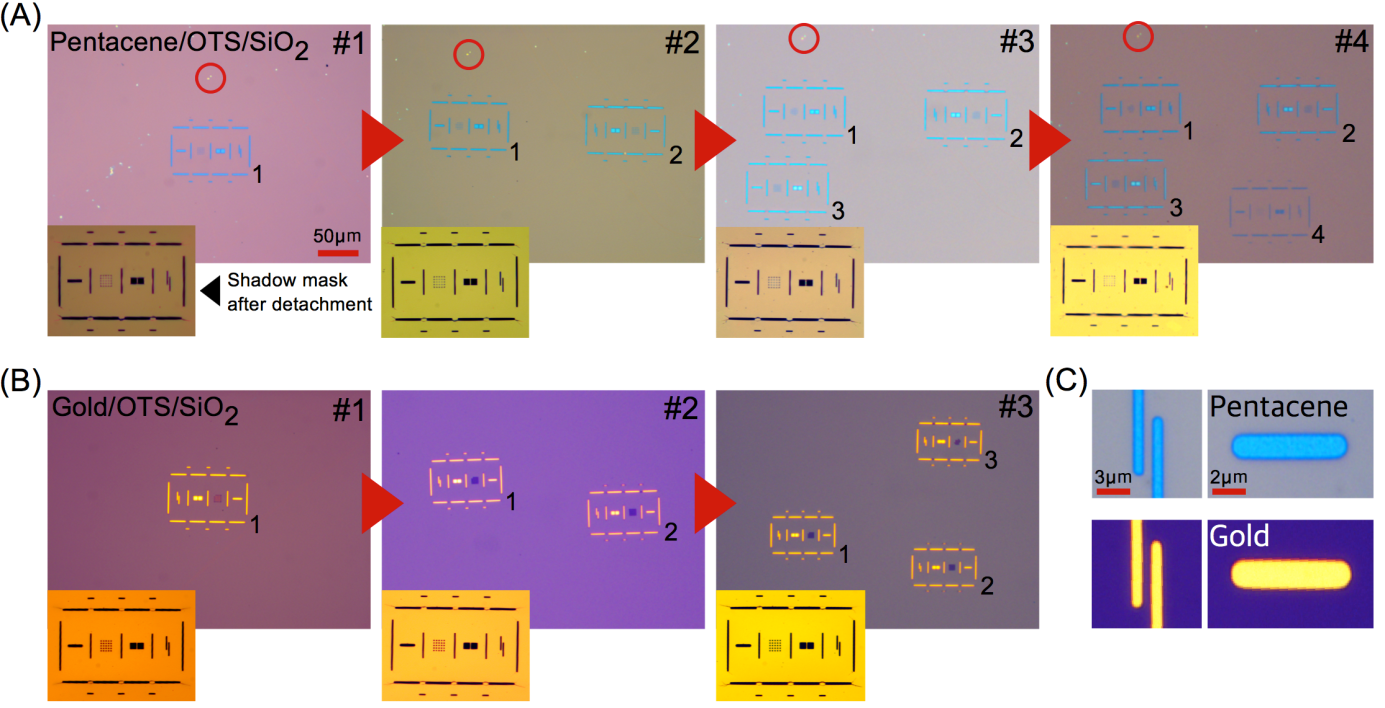


**Supplementary Figure 5**. **The optical microscope images for the reusability test of shadow masks.** (a) The pentacene patterns were repeatedly deposited on a Si/SiO_2_/OTS substrate using only one PMMA mask with four main apertures such as bar, dots, rectangular pads and interdigtated electrodes. The reused PMMA mask was shown under each optical image of deposited pentacene patterns. The red circle indicates impurities on the substrate proving the depositions were carried out on same substrate. (b) Gold layers were evaporated on Si/SiO_2_/OTS substrate by another PMMA mask with same pattern used in (a). (c) Enlarged optical images for pentacene and gold structures deposited through the bar and interdigitated apertures of the PMMA mask.

**Supplementary discussion 1**

**Output characteristics**

Supplementary Figure 1 shows the output characteristics of the pentacene FETs covering the whole range of the prepared channel lengths. The current saturation was clearly shown for longer channel lengths, from 50 µm down to 20 µm. The non-saturation behavior of output curves was gradually observed as the channel length was reduced below 10 µm. In the case of 10 µm, the current value at saturation slightly increased as the bias voltage was increased, which indicates the effect of space charge limited current under the high drain bias condition. For channel lengths of less than 5 µm, the pinch-off was no longer distinct. These trends were gradually enhanced until a channel length of 2 µm. Although the effect of SCLC was apparent for high drain voltage, it is noticeable that the transistor performances such as on/off ratio ~1×10^7^, sub-threshold swing (0.8~1.5 V) and field effect mobility ~1 cm^2^/Vs were maintained. However, in the sub-micron channel regime, the pinch-off and saturation disappeared. For low drain voltages, non-linear behavior was clearly displayed.

**Supplementary discussion 2**

**Pentacene devices with various channel widths**

The field effect mobility was determined as a function of channel width for widths in the range of 100 µm to 300 nm. The width-controlled pentacene FETs were fabricated using the same procedures as described in Fig. 1. Supplementary Figure 2a shows optical images of the channel regions of the fabricated pentacene FETs. The channel length of all devices was fixed to 5 µm since the best device performance was found for a length of 5 µm in the channel-length dependent mobility measurements, as shown in Fig. 5b. The mobility values of all devices were estimated to be around 1 cm^2^/Vs as shown in Supplementary Fig. 2b. These values are consistent with the maximum mobility value obtained in the channel-length dependence experiment. The mobility values are independent of the modulated channel widths, even though the nanoscale channel widths were comparable with the size of a single grain in the pentacene channel.

**Supplementary discussion 3**

**Transfer characteristics and hysteresis behavior**

Representative transfer characteristics of the pentacene FETs in the short channel regime are illustrated in Supplementary Fig. 3. The hysteresis value was defined by taking the threshold voltage difference between forward and backward sweep curves. The forward sweep current was always found to be higher than the backward sweep current for a given gate voltage over the entire range of investigated channel lengths. In previous reports, this hysteresis, with a lower backward sweep current, in pentacene FETs could be explained by the effect of long-lifetime charge traps in the bulk region of the organic semiconductor film or at the interface between the semiconductor and insulator layers. ^[1]^

**Supplementary discussion 4**

**Channel length dependent mobilities of pentacene FETs**

For sub-micron channel lengths, only the linear mobility was estimated since a saturation region was not clearly distinguishable in the output curves so that the gradual channel model could not be directly applied to calculate the field effect mobility from the saturation region. The trend of the channel length dependent linear mobility looks similar to the behavior of the saturation mobility. The mobility value reached a maximum for a 5 µm channel length and decreased as the channel length was reduced to the sub-micron scale. However, it was found that there was less than a factor of two difference in the linear mobility for channel lengths between 5 µm and 50 µm, while the difference in the saturation mobility values for the same range was more than five times. The smaller difference for the linear mobility as a function of channel length could be due to the lower source-drain voltage. In the short channel regime, the magnitude of space-charge limited current (SCLC) is proportional to the square of the longitudinal electric field. Therefore, a much lower SCLC can be expected for lower V_DS_. The linear mobility values were estimated for a source-drain voltage of around -10 V while the saturation mobility values were obtained at a source-drain voltage of -50 V. Thus SCLC can be considered to be the source of the significantly increased saturation mobility under the high bias conditions of the saturation measurements.

**Supplementary discussion 5**

**The reusability of PMMA masks**

The reusability of the PMMA masks was evaluated by repeated depositions of pentacene and gold. As shown in Supplementary Fig. 5a,b, the PMMA masks were successfully reused four times for patterning of pentacene and three times for gold deposition. During the repeated deposition processes, the main apertures of the reused masks were totally maintained without distortion and it was noticeable that the pre-deposited pentacene and gold layers were unharmed by the following deposition steps. The accurate configuration and clear edge of the patterns were reproduced as can be confirmed in Supplementary Fig. 5c showing bar and interdigitated patterns, which were the main structures used to fabricate nanoscale pentacene FETs.

**Supplementary References**

.

1. M. Egginger, S. Bauer, R. Schwödiauer, H. Neugebauer, N. S. Sariciftci, Current versus gate voltage hysteresis in organic field effect transistors, *Monatsh Chem* **140**, 735-750, (2009).
